# Reinvigorating an Academy of Medical Educators Using Ecological Systems Theory

**DOI:** 10.7759/cureus.21640

**Published:** 2022-01-26

**Authors:** Eric Zwemer, Fei Chen, Gary L Beck Dallaghan, Christina Shenvi, Lindsay Wilson, Susan M Martinelli, Morgran Resnick-Kahle, Jason Crowner, Benny L Joyner, Lauren Westervelt, Joanne M Jordan, Alice Chuang, Amy Shaheen

**Affiliations:** 1 Pediatrics, University of North Carolina at Chapel Hill, Chapel Hill, USA; 2 Anesthesiology, University of North Carolina School of Medicine, Chapel Hill, USA; 3 Medical Education, University of North Carolina School of Medicine, Chapel Hill, USA; 4 Emergency Medicine, University of North Carolina School of Medicine, Chapel Hill, USA; 5 Medicine, University of North Carolina School of Medicine, Chapel Hlll, USA; 6 Faculty Affairs and Development, University of North Carolina School of Medicine, Chapel Hill, USA; 7 Surgery, MedStar Union Memorial Hospital, Baltimore, USA; 8 Pediatrics, University of North Carolina School of Medicine, Chapel Hill, USA; 9 Medicine, University of North Carolina School of Medicine, Chapel Hill, USA; 10 Obstetrics and Gynecology, University of North Carolina School of Medicine, Chapel Hill, USA

**Keywords:** school of medicine, bronfenbrenner’s ecological systems theory, academies of medical educators, communities of practice, faculty development

## Abstract

The educational framework of communities of practice postulates that early learners join medical communities as social networks that provide a common identity, role modeling and mentorship, and experiential learning. While being elected into a medical society is an honor, member engagement in these groups can falter if the society membership is seen as an honorific rather than one requiring continuing participation. As an example, Academies of Medical Educators have been established by many academic medical centers to encourage collaboration, skill development, professional identity formation, and scholarship. The University of North Carolina established the Academy of Educators in 2006 to create a diverse community of educators to promote the scholarship, teaching skills, and professional identity of educators. Despite rapid growth to over 500 members, we had less than 30 participants at events over the 2017-2018 academic year. To increase member engagement and participation, our academy leadership team used Bronfenbrenner’s Ecological Systems Theory to design interventions at each layer of environmental influence, specifically at the microsystem, mesosystem, exosystem, macrosystem, and chronosystem levels. In this paper, we describe the multipronged approach used to increase the University of North Carolina Academy of Medical Educators event attendance from 30 to 1,000 faculty participants over the course of one academic year (2018-2019). This paper provides a model as to how medical societies can use ecological systems theory as a natural and comprehensive approach to plan and improve their member engagement and experience.

## Introduction

The educational framework of communities of practice postulates that novices join medical communities as social networks to provide a shared identity, experiential learning, role modeling, and mentorship [1-3]. Such factors allow novices to grow and progress from new members of the group to fully developed members. The experiences necessary for this growth rely upon the active and ongoing engagement of community members at all levels. With the significant competing demands on members of the medical profession, however, engagement in societies can be challenging to maintain. In particular, groups in which members are nominated into membership risk being viewed as solely honorific rather than requiring ongoing participation. As the primary value of such entities is often the community and collaboration, decreasing member engagement can create a domino effect of further and further decreases in event attendance and engagement.

One particularly vulnerable group of faculty is clinician-educators. The Accreditation Council for Graduate Medical Education requires only basic training in teaching skills, but not in more advanced topics such as learning theory, curriculum design, and educational scholarship [4]. As a result, new faculty interested in medical education frequently must navigate their own skill development and promotion along educational pathways. Furthermore, without clearly configured support systems at the departmental and institutional level, faculty members interested in medical education are often separated into field-specific silos, leading to duplication of efforts and missed opportunities for inter-disciplinary engagement.

To encourage collaboration, skill development, professional identity formation, and scholarship, some academic medical centers have created academies of medical educators (AMEs) [5]. Over the past two decades, AMEs have grown significantly in size and number across the United States. Currently, 72 AMEs are members of the Association of American Medical Colleges Academies Collaborative [6]. While definitions vary with respect to the structure, membership, and content of AMEs, the success of an AME can vary from site to site [5].

The University of North Carolina (UNC) School of Medicine established the Academy of Educators in 2006 to create a diverse community of educators to promote the scholarship, teaching skills, and professional identity of educators. Despite rapid growth to over 500 members, we had less than 30 participants at our sponsored events over the 2017-2018 academic year. The cultural perception among faculty was that our Academy was an honorific society rather than an actively engaged group of educators. In May 2018, we sought to reinvigorate our Academy of Educators through a multi-pronged approach applying Bronfenbrenner’s framework.

Bronfenbrenner’s Ecological Systems Theory [7,8] characterizes multiple environmental layers that affect human development. These environments range from the microsystem (immediate environment) to the macrosystem (social and cultural values) to the chronosystem (changes over time). While these environments are interrelated, each can also be thought of as a separate tier of influence on an individual's development. This theory has been extensively applied to human development, K-12, and higher education. Recently, Hamwey et al. [9] used this model to describe the different impacts of the environment on the learner. In this paper, we discuss the strategies used to address each layer of Bronfenbrenner’s model, which resulted in increased member engagement to over 1,000 participants at AME events over one year. Although our example is situated within a school of medicine, this model can easily be applied to other interdisciplinary societies and community organizations.

## Technical report

Microsystem

The microsystem consists of the interpersonal interactions, human relationships, and the immediate environments surrounding a faculty member.

Offer In-Person Events at Regional Sites

Our large faculty population is distributed across the state, which includes six Area Health Education Center (AHEC) campuses. In the past, we have struggled to maintain participation and engagement among these regional campuses. As a result, we began offering programs at the other campuses facilitated by central and AHEC faculty. This in-person experience fostered connection between the campuses and the AME, consequently improving faculty engagement. In one year, almost half of our total sessions took place at these regional campuses. We offered two sessions at each of our five campuses.

Mesosystem

The mesosystem refers to interactions and linkages that occur between two or more microsystems. Our AME reinvigoration sought to specifically affect the mesosystem to improve mentoring of early-career faculty and collaboration across educational centers.

Add a Mentored Member Pathway for Junior Faculty

While individual departments have mentoring programs, these are variable and frequently focus on clinical work or biomedical research. To address this gap, we created a mentored member program for faculty who had been part of our institution for two years or less. Applicants for mentored membership were selected based on their prior accomplishments in education, dedication to education, and potential for future educational success. In the first year of the program, 20 individuals were selected and received professional development on how to succeed as educators in academic medicine. The mentored member group created a network of highly promising and engaged inter-disciplinary junior faculty. As they progress through their careers, we anticipate that they will be able to collaborate with their colleagues across departments. This process has specifically served to connect young faculty within individual departments (i.e., one microsystem) both to faculty mentors outside of their department and to each other (i.e., two additional microsystems).

Designate Regional Site Liaisons

Every healthcare location has its own culture and organization regarding best venues and timing for educational sessions, existing local educational resources, and key contacts for educational opportunities. To better serve faculty at all campuses, we created AME Faculty Champions at each AHEC site. These individuals have an existing role in education at their campus, giving them perspective on the available resources and needs of faculty. The interaction of these Champions with the central AME committee leads to increased and improved AME offerings to AHEC faculty. One comment from an AHEC faculty leader at a SOM retreat summed up the effect of the Champions, “[AME] is relevant to us for the first time.”

Exosystem

The exosystem refers to linkages between two or more settings, at least one of which is indirect to the faculty member. We focused our efforts on firmly linking SOM leadership to the AME and enabling virtual connections.

Increase Resources and Supports Within the School of Medicine

While the SOM had long provided financial support for the AME, frequent interactions and personnel support were limited. Early in our first year, AME council members met regularly with SOM deans, departmental leaders, and the SOM development office. The development office committed an additional annual budget of $15,000 for AME grants for educational innovations and research. These developments did not directly involve our faculty members at large but led to extensive beneficial programmatic changes and scholarly offerings. In addition to the money allocated from the development office, the SOM increased the AME budget by 9% for the upcoming year to create and implement new faculty development programs for educators. The SOM providing financial support for this programming benefits all faculty in the SOM and encourages a continued mutually beneficial relationship between the AME and SOM.

Enable Virtual Connections and Inclusions

To further involve faculty who were unable to attend events in person, we recorded and live-streamed programs occurring on the main campus. Faculty now have the option to watch the sessions at their own convenience if their schedule does not allow for synchronous participation. These options improved the participation of our faculty, both at other campuses and those on the main campus whose schedules made it difficult to attend in person. Over the course of 2018-2019, we had 116 faculty participants that viewed events through distance-learning options, and recordings of workshops were viewed a total of 34 times.

Macrosystem

The macrosystem involves cultural norms and values imposed by society and experienced by individual faculty members. Changes to cultural norms and values can be the most difficult to achieve but often represent the most important and sustainable changes. We sought to specifically change the perception of the AME, raise the stature of education and educators, and create a culture of engagement and interaction.

Promote a Culture of Active Engagement

Historically, election into the AME required two years on the faculty, a clear dedication to education, and two nominating letters. While reapplication was required every five years, denial of an application was rare. The perception arose among many departments that the AME was purely an honorific society that required no active participation. For the 2018-2019 academic year, we required AME members to attend at least two events annually to maintain their membership. To help them achieve this minimal number, we offered almost 25% more events per year, held events across multiple sites, offered virtual options for attendance, and allowed asynchronous video review of sessions. For the 2019-2020 year, the membership voted to increase the membership requirement for attendance to four events per year, suggesting a desire for a more involved cohort. Furthermore, continued membership is contingent upon demonstrated participation in AME activities, not educational expertise. Building the desired participation in AME events into the qualifications for ongoing membership serves to promote the culture of the AME as an active organization.

Focus on Workshops Over Didactics

The key to the success of any faculty development organization is the quality of the educational events themselves. As noted above, prior AME events had consisted of primarily didactic sessions. For adult learners, faculty development sessions should be relevant and engaging in a non-threatening environment [10]. Mccoy et al. [11] identified five conditions for effective learning: real-world relevance, competency-based, collaborative, deliberate practice, and technology-enhanced. With this in mind, we deliberately restructured and renamed our 2019-2020 sessions “workshops,” emphasizing that attendees would leave sessions with a skill or product applicable to their work. Such sessions maintain a didactic component, limited to 20-30 minutes, while the remainder of the 60-minute session consists of deliberate faculty practice and discussion. Over the past year, attendees have designed their own teaching scripts for physical examination, practiced an educational “story” to use with trainees, and envisioned the “hook” to start their next talk. This focus on relevance and deliberate practice led to highly engaging sessions.

Celebrate Membership Achievements

To increase the perceived institutional value of education and educators, we sought to increase the recognition of AME member achievements. First, we submitted AME events to feature in the weekly institution-wide newsletter, advertising the valuable contributions of our members. Second, we highlighted a member each quarter on our website, including a brief biography and interview focusing on their educational interests. Lastly, we revised our annual AME faculty awards to create additional awards for graduate medical educators. Unintentionally, these awards focused more on undergraduate medical educators than graduate medical educators, excluding a significant portion of members who have few medical student interactions.

Share the Responsibility and Work

Several missed opportunities existed with the prior small leadership group. First, fewer members were engaged and able to lead projects and initiatives, and the culture became one in which two to three people performed all the work. Second, only a few individuals were able to develop and demonstrate their educational leadership skills and vision. The original leadership system had existed since AME’s inception due to momentum rather than practicality. Given the many responsibilities, it was clear that a larger leadership council was needed. With only a slight increase of 5% funding for individual faculty salary support, we reallocated the support to a broader faculty group, transitioning to a council consisting of a President and six individual committee chairs elected by the membership. In addition to committee participation, each council member led a specific domain that allowed for increased work products, evidenced by increased offerings at our main and AHEC sites, the transition of events from didactics to workshops, and the creation of new programming options. We also engaged more individuals in leadership by allowing AME members to serve as committee leaders and participants. We solicited members to participate in task forces that address membership, teaching awards, and grant reviews. In this way, members could invest in the AME and demonstrate leadership capability and service to the institution, which can be considered in their promotion pathway.

Involve Non-Physician Educators

While many of our faculty involved in education hold PhDs rather than medical degrees, the leadership of the AME was traditionally by physicians. The vast majority of our members are healthcare providers dedicated to education for the eventual goal of improving patient care; however, the variance in the capability and training in the educational scholarship of the group is large. In particular, we found that the experience and skills of our faculty in educational scholarship were lacking, with few physician role models who had the time and expertise to advise and train them. Even amongst our original leadership council, only two of the original seven members held advanced degrees in educational scholarship. To address this gap, our academy made the deliberate decision to include two scholars who have doctoral degrees in educational fields and have been prolific in medical education scholarship as members of the leadership council. These non-physician educators provide our leadership council and faculty access to valuable guidance on improving local educational practices and pivotal resources in promoting scholarship. In the 2018-2019 academic year, our education scholarship leads collaborated with AME members on 32 manuscripts.

Chronosystem

The final component of Bronfenbrenner’s Ecological Systems Theory is the chronosystem, which describes how changes and transitions in the environment over time will naturally affect development.

Respond to Changing Needs Proactively

The chronosystem incorporates broad-based circumstances and changes that will affect our educators’ needs and desires. These include changes in leadership, promotion processes, and financial distributions of the SOM. These events may shift the SOM’s educational priorities and lead to downstream effects on individual educators. Additionally, as our learners’ needs and desires shift, the needs and desires of our educators also change. Periodic needs assessments allow the AME to identify and respond to these changing needs in a timely manner. Information on preferred timing of events, the format of presentations, and desired topics allowed us to deliver more relevant and accessible content. We also surveyed department chairs asking about educational and scholarship resources offered by individual departments. Our goal is to share the collected information with chairs to encourage them to standardize and increase educational resources. Finally, in addition to bimonthly meetings of the leadership council and sub-committees, we instituted the Annual Strategic Planning Retreat for Education, a yearly retreat for AME leadership. The retreat focused on the overall mission and vision of the AME, allowing for more creative and global thought and discussion, as compared to the details and urgent tasks that frequently fill the agenda for our bimonthly meetings.

## Discussion

In this paper, we detailed several strategies in the successful reinvigoration of our AME over one academic year (Table 1). As a framework for understanding the multiple environmental levels that affect an individual faculty member’s experience in the AME, we utilized Bronfenbrenner’s ecological systems theory to guide the plan and enact changes. As these changes occurred, we saw notable success in overall attendance at AME events, involvement in AME activities, and expansion of AME programmatic offerings. Notably, these changes occurred pre-pandemic and represent ways of thinking about faculty engagement even outside of the current COVID-19 pandemic. We do not suggest that each of the above specific strategies and consequential changes will be applicable to every institution or organization, but rather than basing strategies on an ecological systems framework provides a comprehensive and sustainable approach to plan and improve a society’s experience for faculty.

**Table 1:** Overview and application of Bronfenbrenner’s ecological systems theory to the University of North Carolina School of Medicine Academy of Medical Educators

System Level	Definition	AME Strategy/Approach
Microsystem	Human relationships, interpersonal interactions, and the immediate environment surrounding a faculty member (i.e., hospital, clinic, or school environment).	Offer in-person programming at all sites
Mesosystem	Interactions and linkages between 2 or more microsystems	Add a mentored member pathway for junior faculty
		Designate regional site liaisons
Exosystem	Linkages and processes between 2 or more settings, at least one of which is indirect to the learner, both of which the impact learner	Increase resources and support within the School of Medicine
		Enable virtual connections and inclusion
Macrosystem	Cultural norms and values imposed by society and experienced by individuals. Changes to cultural norms and values can be the most difficult to achieve, but often represent the most important and sustainable changes.	Promote a culture of active engagement
		Focus on workshops over didactics
		Share the responsibility and work
		Celebrate membership achievements
		Involve non-physician educators
Chronosystem	Patterning of events and transitions over life course	Respond to changing needs proactively

## Conclusions

Medical societies may struggle with limited faculty engagement over time and risk membership being viewed as honorific rather than requiring active participation. While many possible approaches to these problems exist, AMEs and similar faculty organizations from other institutions may benefit from the application of Bronfenbrenner's Ecological Systems Theory to their efforts to improve faculty involvement and retention. This paper provides a model as to how medical societies can use ecological systems theory as a natural and comprehensive approach to plan and improve their member engagement and experience.
